# Clinical effect of Lymph Node Dissection in the Lateral Neck for the treatment of Papillary Thyroid Carcinoma: Endoscopic Thyroidectomy versus Open Surgery

**DOI:** 10.12669/pjms.39.6.7156

**Published:** 2023

**Authors:** Guangyu Wang, Gang Zhang, Xiaoming Gao

**Affiliations:** 1 Guangyu Wang, Department of General Surgery, Baoding No.1 Central Hospital, Baoding, 071000, Hebei, China; 2 Gang Zhang, Department of General Surgery, Baoding No.1 Central Hospital, Baoding, 071000, Hebei, China; 3 Xiaoming Gao, Department of General Surgery, Baoding No.1 Central Hospital, Baoding, 071000, Hebei, China

**Keywords:** Papillary thyroid carcinoma, Endoscopic-assisted, Lymph node dissection, Oxidative stress, Cosmetic effect

## Abstract

**Objective:**

To compare the clinical effect, safety, and cosmetic effect of lymph node dissection via endoscopic thyroidectomy and open surgery in the treatment of papillary thyroid carcinoma (PTC).

**Methods:**

A retrospective analysis was performed on 86 patients with PTC admitted to Baoding No.1 Central Hospital from January 2020 to December 2021. The enrolled patients were divided into the endoscopic thyroidectomy (n = 34) and open surgery (n = 52) groups based on different surgical methods. Further comparison was conducted on the surgical indexes, postoperative complications, changes of oxidative stress indexes, numerical score system (NSS) score of postoperative cosmetic satisfaction, scar length, and recurrence rate between the two groups.

**Results:**

The operation time was longer, and the intraoperative blood loss was lower for the endoscopic thyroidectomy group than those for the open surgery group (*P* < 0.05). The oxidative stress indexes of the endoscopic thyroidectomy group were significantly better than those of the open surgery group (P< 0.05). The postoperative complication rate of the endoscopic thyroidectomy group was lower than that of the open surgery group (*P* < 0.05). At six months after surgery, the scar length was shorter, and the NSS score was higher in the endoscopic thyroidectomy group than those in the open surgery group (*P* < 0.05).

**Conclusion:**

Endoscopic thyroidectomy for PTC is safe and effective, with mild stress response, fewer postoperative complications, and good cosmetic effect.

## INTRODUCTION

Thyroid cancer is a common malignant tumor of the endocrine system, and its incidence has increased continuously in recent decades.^1^ Papillary thyroid carcinoma (PTC) is the most common type of thyroid cancer and accounts for 80-90% of the incidence rate of thyroid cancer.^2^,^3^ Traditional open surgery is still the major therapeutic choice for PTC; however, this treatment may result in a long length of incision, strong postoperative stress reaction and formation of neck surgical scars after surgery.^4^ With the continuous development of minimally invasive technology and the increasing demand for beauty, the endoscope has been extensively applied in surgery, and endoscopic thyroidectomy has rapidly developed.

Compared with open surgery, endoscopic surgery has the advantages of small incisions, minimal invasiveness, and cosmetic effect.^5^ At present, endoscopic thyroidectomy has only been clinically applied for a short period of time. Additional evidence-based medical evidence is needed in clinical practice to verify the surgical safety, feasibility, advantages, and disadvantages of endoscopic thyroidectomy for PTC and to guide clinical work. Through a retrospective approach, this study evaluated the clinical application effect of endoscopic thyroidectomy for PTC and provided a clinical reference for the minimally invasive treatment of PTC.

## METHODS

A retrospective analysis was carried out on the clinical data of 86 patients with PTC confirmed by surgery and pathology who were admitted to Baoding No.1 Central Hospital from January 2020 to December 2021. According to different surgical methods, the eligible patients were divided into endoscopic thyroidectomy (n = 34) and open surgery (n = 52) groups.

### Ethical Approval

This study was approved by the Institutional Ethics Committee of Baoding No.1 Central Hospital (No.: [2022]059); dated: August 03, 2022). Owing to the retrospective design of this study, individual informed consent was waived.

### Inclusion criteria:

- Patients who met the diagnostic criteria of PTC in the American Thyroid Association guidelines.^6^
- Patients with initial onset without previous history of neck radiotherapy, ablation, or other surgical treatments.
- Patients with tumor diameter ≤3 cm.^7^
- Patients with metastatic lymph nodes ≤2 cm in diameter.
- Patients without other systemic malignancies or surgical contraindications.
- Patients with complete clinical data.

### Exclusion criteria:

- Patients with distant diffusion of tumor cells.
- Patients combined with other malignant tumors.
- Patients with treatment converted from endoscopic thyroidectomy to open surgery.
- Patients with severe thyroid dysfunction.

Open surgery group: An arc-shaped incision of about 12-15 cm in length was made along the dermatoglyph 1.5-2.0 cm above the anterior sternal notch of the neck, which was biased toward the side of lymph node dissection. After the layer-by-layer incision, total thyroidectomy + lymph node dissection in the central region or unilateral thyroidectomy and isthmectomy + lymph node dissection in the central region of the affected side were performed. During the operation, the enlarged lymph nodes and soft tissues in regions-II, III, and IV; moreover, the medial internal jugular vein were completely removed.^8^

A drainage tube was placed, and the incision was sutured layer by layer after hemostasis. Endoscopic thyroidectomy: Endoscopic-assisted lymph node dissection in the lateral neck was performed, and an incision of 5-7 cm in length was made above the anterior cervical sternal notch of the anterior neck. Thyroid and central lymph nodes were treated in the same way as in the open surgery group. Endoscopic instruments were then placed into the anterior cervical incision. The enlarged lymph nodes and soft tissues in regions II, III, and IV and the medial internal jugular vein were completely removed.^9^ The remaining operation steps were continued the same as those in the open surgery.

(1) Operation-related indicators included operation time, intraoperative blood loss, number of lymph nodes dissected, number of positive lymph nodes, postoperative drainage time, and postoperative length of stay. (2) Surgical oxidative stress-related indexes included specific indicators, such as glutathione peroxidase (GSH-Px), superoxide dismutase (SOD), and malondialdehyde (MDA) levels. (3) Postoperative complications^10^ included choking when drinking, transient hoarseness, hypoparathyroidism, hypocalcemia and postoperative hematoma. (4) Postoperative cosmetic effect and recurrence: The scar length was measured six months after the operation. The patient’s satisfaction with the incision scar was evaluated by the patients themselves using the numerical score system (NSS).^11^ The score ranged 0-10 points: a high score indicates that the patients have a high degree of satisfaction. The recurrence and metastasis rates of the two groups were calculated every six months.

### Statistical analysis

SPSS 26.0 statistical software was used for analysis. The measurement data were expressed by means ± standard deviation (*χ̅*±*S*) and compared between groups using a t-test. The counting data were expressed in n (%) and compared between groups by χ^2^ test. *P* < 0.05 indicated that the difference was statistically significant.

## RESULTS

No significant difference in general data was observed between the two groups (*P* > 0.05), suggesting comparability between them, as shown in Table-I. The operation time of the endoscopic thyroidectomy group was longer than that of the open surgery group, but the intraoperative blood loss of the former was lower than that of the latter (*P* < 0.05), as shown in Table-II.

**Table-I:** Comparison of general data between the two groups (*χ̅*±*S*).

Items	Endoscopic thyroidectomy group (n=34)	Open surgery group (n=52)	t/χ^2^	P
Gender (male/female)	3/31	10/42	1.735	0.188
Age (years)	31.41±7.35	33.92±7.27	1.560	0.123
Surgery mode (n,%)			0.062	0.803
Total thyroidectomy	12 (35.29)	17 (32.69)		
Unilateral thyroidectomy	22 (64.71)	35 (67.31)		
Side of dissection (n,%)			5.403	0.067
Left	5 (14.71)	13 (25.00)		
Right	10 (29.41)	23 (44.23)		
Bilateral	19 (55.88)	16 (30.77)		

**Table-II:** Comparison of operation related indexes between the two groups.

Items	Endoscopic thyroidectomy group (n=34)	Open surgery group (n=52)	t/Z	P
Operation time (min)	172.59±44.02	137.15±31.85	4.329	0.000
Blood loss (ml)	47.65±10.09	60.48±13.37	4.775	0.000
Postoperative length of stay (d)	6.00(3.00)	6.00(1.75)	0.673	0.501
Number of lymph nodes dissected (n)	39.00(20.75)	37.50(23.50)	1.167	0.247
Number of positive lymph nodes (n)	9.00(8.50)	7.00(6.75)	0.316	0.753
Postoperative drainage time (d)	2.00(1.50)	2.00(1.00)	0.977	0.332

At 72 hours after the operation, the GSH-Px and SOD levels in both groups were significantly lower than those before the operation, and the MDA levels were significantly higher than those before the operation (*P* < 0.05). Table-III. Statistically significant differences in the above indexes were found between the two groups at the corresponding time points (*P* < 0.05).

**Table-III:** Comparison of oxidative stress-related indexes between the two groups (*χ̅*±*S*).

Groups	GSH-Px (mg/L)		SOD (u/ml)		MDA (u/ml)	

	Before treatment	After treatment	Before treatment	After treatment	Before treatment	After treatment
Endoscopic thyroidectomy group	149.59±3.97	130.18±5.10*	116.24±3.98	60.26±4.20*	7.05±1.57	9.91±1.40*
Open surgery group	148.45±3.20	127.06±2.22*	115.65±6.25	55.12±4.21*	7.35±0.98	11.13±1.23*
*t*	1.470	3.891	0.482	5.547	1.087	4.286
*P*	0.145	0.000	0.631	0.000	0.280	0.000

***Note:***

* compared with the level before treatment, *P*<0.05.

Hematoma was recorded in six patients in the open surgery group and none in the endoscopic thyroidectomy group, with a statistically significant difference found between the two groups (*χ^2^* = 4.217*, P* < 0.05). Ten patients in the endoscopic thyroidectomy group and 27 patients in the open group developed complications. The postoperative complication rate of the endoscopic thyroidectomy group (29.41%) was lower than that of the open group (51.92%) (*χ^2^* = 4.250*, P* < 0.05), as shown in Table-IV.

**Table-IV:** Comparison of postoperative complications between the two groups (n,%).

Groups	Hoarseness	Hypoparathyroidism	Hypocalcemia	Postoperative hematoma	Total
Endoscopic thyroidectomy group (n=34)	5(14.71)	6(17.65)	5(14.71)	0(0.00)	10(29.41)
Open surgery group (n=52)	8(15.38)	11(21.15)	9(17.31)	6(11.54)	27(51.92)
*χ^2^*	0.007	0.159	0.102	4.217	4.250
*P*	0.932	0.690	0.749	0.040	0.039

At six months after surgery, the scar length in the endoscopic thyroidectomy group was shorter than that in the open surgery group, and the NSS score was higher in the former than in the latter (*P*< 0.05, Table-V). No significant difference in recurrence rate and metastasis rate was observed between the two groups at the 6th-month follow-up (*P* > 0.05, Table-VI).

**Table-V:** Comparison of postoperative cosmetic effects between the two groups (*χ̅*±*S*).

Groups	Scar length (cm)	NSS(Score)
Endoscopic thyroidectomy group (n=34)	6.00±0.70	6.50±1.05
Open surgery group (n=52)	11.81±0.99	3.31±0.92
*t*	29.690	14.871
*P*	0.000	0.000

**Table-VI:** Comparison of short-term recurrence and metastasis between the two groups (n,%).

Groups	Recurrence	Metastasis		

		Bone metastasis	Lung metastasis	Total
Endoscopic thyroidectomy group (n=34)	4 (11.76)	2 (5.88)	2 (5.88)	4 (11.76)
Open surgery group (n=52)	3 (5.77)	1 (1.92)	2 (3.85)	3 (5.77)
*χ^2^*	0.988	0.957	0.192	0.988
*P*	0.320	0.328	0.661	0.320

## DISCUSSION

In our study, the endoscopic thyroidectomy group had longer operation time but less intraoperative blood loss than the open surgery group, with statistically significant differences. This finding was consistent with previous reports. In terms of surgical safety, the postoperative complication rate of the endoscopic thyroidectomy group was lower than that of the open surgery group. This result suggested that endoscopic thyroidectomy is superior to open surgery in safety and can reduce surgery-related discomfort in patients.

As an invasive treatment, surgery can negatively affect the homeostasis of the internal environment, leading to oxidative stress that may disturb thyroid function. GSH-Px and SOD are the most common reactive factors in oxidative stress.12 GSH-Px is a peroxidase that can enhance tissue antioxidant capacity.13 MDA is an important biomarker of oxidative stress that can induce the formation of free radicals and increase the degree of secondary damage. In this study, postoperative measurements revealed that the values of the above indicators were better in the endoscopic thyroidectomy group than in the open surgery group. This finding indicated that endoscopic thyroidectomy can effectively reduce oxidative stress reaction compared with traditional open surgery, which may be explained by the reduced traumas from the former.

Thyroid cancer is relatively common clinically nowadays, and its incidence rate is increasing year by year. Papillary thyroid carcinoma(PTC) is the major histological type of thyroid cancer, with middle-aged and young women comprising the high-risk population.^14^ The main treatment option for thyroid cancer is surgery, which generally includes thyroidectomy and neck lymph node dissection.^15^ Traditional open surgery is a mature technical procedure for the treatment of thyroid cancer but has disadvantages, such as great trauma, slow recovery, and many complications.^15^,^16^ At present, minimally invasive endoscopic surgery is widely clinically applied in surgical fields. With a small incision in the neck, this surgical technique is completed with the use of an endoscope and ultrasonic knife.

A previous study found no difference in clinical effect and safety between endoscopic surgery and traditional open surgery.^17^ Moreover, the former technique has the advantages of small incisions, inconspicuous scars, and good cosmetic effects. However, the safety and clinical effect of endoscopic surgery on the treatment of thyroid cancer remains controversial.^18^

Despite the requirement for prolonged operation time owing to the relatively complex and complicated surgical procedure, endoscopic surgery is a minimally invasive operation with hemostasis and resection during the operation and is associated with a relatively small amount of intraoperative bleeding.

Postoperative scarring after thyroidectomy is an important problem for thyroid cancer patients, especially female patients. The formation of surgical scars can be attributed to multiple factors. The surgical incision for thyroid cancer is located in the neck. Through traditional open surgery, the dissociation of thyroid glands requires a large field of surgery and hence a large incision, leading to postoperative scar hyperplasia.^19^ Generally, surgical scars begin to mature six months after surgery. In our study, the surgical scars of the enrolled patients were evaluated six months after the operation. The results showed that the patients who underwent endoscopic thyroidectomy for PTC had short postoperative scars. Therefore, this operation can meet the needs of patients in terms of aesthetics. The conclusion of this study provides a clinical reference for the minimally invasive treatment of PTC.

### Limitations of this study

The findings require further validation based on randomized controlled studies due to the small sample size and short follow-up of the present work.

## CONCLUSION

For the treatment of lateral neck lymph node metastasis in patients with PTC, endoscopic thyroidectomy for lymph node dissection in the lateral neck has the advantages of reducing intraoperative bleeding, postoperative complications, and oxidative stress response and achieving a good cosmetic effect.

### Authors’ Contributions:

**GW** carried out the studies, participated in collecting data, and drafted the manuscript, and are responsible and accountable for the accuracy or integrity of the work.

**GZ** performed the statistical analysis and participated in its design.

**XG** participated in acquisition, analysis, or interpretation of data and draft the manuscript. All authors read and approved the final manuscript.
